# Integration of substrate-specific enzymes and a peroxide biosensor for detection of glucose, uric acid, and cholesterol

**DOI:** 10.1128/aem.00338-26

**Published:** 2026-05-18

**Authors:** Mengmeng Ji, Hong Sun, Guifang Shi, Wei E. Huang, Yun Wang

**Affiliations:** 1 Oxford Suzhou Centre for Advanced Research (OSCAR), University of Oxford, Suzhou, Jiangsu, People's Republic of China; 2 Department of Endocrinology and Metabolism, Suzhou Dushu Lake Hospital, The Fourth Affiliated Hospital of Soochow University, Medical Center of Soochow University 606537, Suzhou, Jiangsu, People's Republic of China; 3 Department of Engineering Science, University of Oxford 6396, Oxford, United Kingdom; Kyoto University, Kyoto, Japan

**Keywords:** biosensor, hydrogen peroxide, enzyme coupling, glucose, uric acid, cholesterol

## Abstract

Conventional whole-cell biosensors function as “one analyte, one sensor” systems to ensure high specificity relying on transcriptional regulatory protein cascades to induce reporter gene expression, leading to the development of distinct sensor systems to monitor multiple target substances. To address this constraint and broaden analyte detection capabilities, we developed a modular biosensing platform that couples a key metabolite-responsive biosensor with various substrate-specific enzymes to detect a range of target molecules. This study demonstrated that the integration of a general hydrogen peroxide (H_2_O_2_)-responsive biosensor with glucose oxidase, uricase, and cholesterol oxidase enabled the detection of three clinically relevant biomarkers: glucose, uric acid, and cholesterol. Upon substrate-specific enzyme conversion, these three biomarkers generate H_2_O_2_, which triggers the expression of the *lux*CDABE reporter gene cluster and produces bioluminescence in the H_2_O_2_-responsive biosensor *Pseudomonas putida* KT2440[pPahpc]. The system has demonstrated sensitive detection of glucose (10–200 µM), uric acid (5–125 µM), and cholesterol (1.25–100µM). Validation with 17 clinical urine specimens confirmed the reliability of our system for quantifying glucose and uric acid, thereby establishing its potential diagnostic utility.

**IMPORTANCE:**

The increasing public focus on health management has fueled the development of decentralized diagnostic testing. While biosensors are ideally suited to this application area, their dependence on molecular specificity mandates individualized transducers for each target. Consequently, multiplied engineering efforts and resource expenditure substantially restrict practical deployment. This study addresses this challenge through the integration of a universal signal transduction pathway with multiple substrate-specific oxidases, enabling simultaneous quantification of diverse molecular targets within a single biosensing platform. Clinical validation with 17 urine specimens confirmed the system’s robust analytical performance. Our findings establish a technological foundation for cost-effective, rapid, and multiplexed home-testing devices, showing substantial promise for advancing disease surveillance and personalized healthcare management.

## INTRODUCTION

With the improvement of living standards, the incidence of metabolic diseases such as diabetes, cardiovascular disease, and hyperuricemia is gradually increasing in modern society, posing an increasingly severe threat to people’s health. Diabetes mellitus, pathophysiologically manifested by insulin dysregulation (1), and subsequent hyperglycemia (2), can escalate to multisystem complications including nephropathy, retinopathy, cardiovascular abnormalities, cerebrovascular incidents, and ultimately fatal organ failure if left untreated (3). Hypercholesterolemia stands as a pivotal etiological contributor to the onset of both atherosclerotic cardiovascular disease and cerebrovascular incidents (4, 5), while the concentration of uric acid (UA) functions as a clinically substantiated indicator for chronic kidney disease progression and gout (6, 7). Currently, the detection of biomarkers for these three diseases—glucose, cholesterol, and uric acid—primarily relies on blood samples, which is an invasive procedure and necessitates specialized equipment (8–10). With the global burden of metabolic disorders continuing to rise, there is an increasing need for real-time, non-invasive monitoring approaches that can facilitate earlier clinical intervention and improved disease management.

Whole-cell biosensors use microbial cells as sensing devices to convert target analytes into measurable signals, such as light, electricity, fluorescent proteins, and changed color (11–13). The intensity of these signals is positively or negatively correlated to the concentrations of the target analytes, thus enabling the quantitative and dynamic monitoring inside or outside of the cell (14). Biosensors offer rapid response, convenience, low cost, small sample volume requirements, sensitivity, and semi-quantitative detection at the nanomolar to millimolar (nM to mM) level. The unique advantages of biosensors, including bioavailability and the ability to perform *insitu* measurements (15), have led to their wide application in biological manufacturing, environmental monitoring (16, 17), food safety (18), medical diagnostics, and therapy (19, 20). Several biosensing platforms have been developed to non-invasively quantify glucose and uric acid in alternative biofluids, such as urine, sweat, and saliva, using advanced microfluidic interfaces (21–24). Nevertheless, most existing whole-cell biosensors depend on native transcriptional regulatory systems that are directly induced by the target analyte, substantially limiting the diversity of detectable molecules.

**Fig 1:**
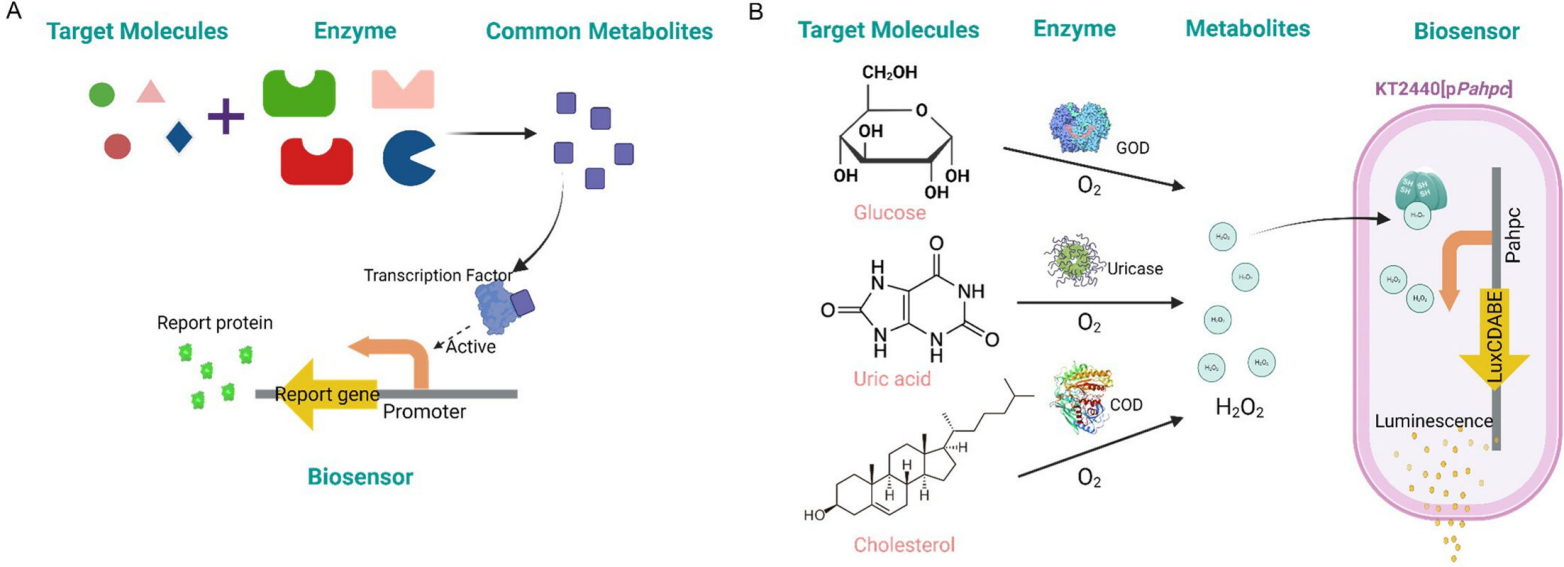
Schematic for multiplexed small molecule detection via a whole-cell biosensor. (**A**)Target analytes are enzymatically converted into a common metabolite by specific enzymes. The shared product enables single-biosensor detection of diverse molecules via a whole-cell system. (**B**)In this proof-of-concept model, KT2440[pPahpc] detects H_2_O_2_ generated from the sequential oxidation of glucose, uric acid, and cholesterol by glucose oxidase (GOD), uricase, and cholesterol oxidase (COD), respectively, illustrating multitarget compatibility.

To overcome this constraint, we proposed a new method in which diverse target analytes are enzymatically converted into a common detectable metabolite, subsequently activating a biosensor (Fig. 1). This approach decouples biosensing from analyte-specific transcription factors, substantially expanding the accessible detection landscape and facilitating scalable reconfiguration of biosensors.

Hydrogen peroxide (H_2_O_2_), a ubiquitous oxidative byproduct of catalytic oxidation processes, serves as a universal enzymatic reporter molecule across multiple biochemical pathways (25, 26). This biochemical mechanism is typified by (i) GOD(EC 1.1.3.4)-catalyzed conversion of β-D-glucose to D-glucono-δ-lactone with stoichiometric H_2_O_2_ generation, (ii) uricase (EC 1.7.3.3)-mediated oxidation of uric acid to allantoin and CO_2_, accompanied by equimolar H_2_O_2_ production, (iii)COD(EC 1.1.3.6)-dependent oxygenation of cholesterol to 4-cholesten-3-one, coupled with enzymatic H_2_O_2_ liberation. Consequently, quantification of enzymatically generated H_2_O_2_ provides a unified transduction strategy for the multiplexed determination of glucose, uric acid, and cholesterol concentrations via standardized enzymatic cascades (27). Various H_2_O_2_ quantification methods, such as spectrophotometry, chromatography, and fluorometry, exhibit analytical validity (28–30). However, their clinical utility is limited by prolonged assay durations (>30min), technical complexity (requiring skilled personnel), and significant infrastructure investments.

Whole-cell biosensors offer a promising alternative. Gao et al. developed the highly sensitive *Pseudomonas putida* KT2440[pPahpc] H_2_O_2_ biosensor (31), which showed high sensitivity and rapid response. Building on this framework, we combined GOD, uricase, and COD with the KT2440[pPahpc] biosensor to construct an integrated detection platform capable of measuring glucose, UA, and cholesterol through a H_2_O_2_-mediated signaling cascade. Urine was selected as the sampling matrix for glucose and UA owing to its non-invasive collection, whereas cholesterol was excluded because of its negligible urinary presence. The resulting multiplexed system, termed the OxChain Biosensor Kit, enables rapid, real-time, and modular detection of metabolic biomarkers using a unified enzymatic reporting strategy.

## MATERIALS AND METHODS

### Chemicals and reagents

(D+)-glucose was purchased from Hushi (Sinopharm, China). Thirty percent hydrogen peroxide (H_2_O_2_) was purchased from Amresco (USA). Agar, UA, cholesterol water-soluble powder, and GOD(224,890 U/g) were obtained from Sigma-Aldrich (USA), and uricase (500 U) and COD(200 U) were purchased from Solarbio (China). The antibiotic tetracycline hydrochloride was obtained from Phyto Tech (USA). Luria-Bertani (LB) (Hopebio, China) or minimal medium was prepared for bacterial cultivation. Minimal medium preparation (1L): 2.5 g Na_2_HPO_4_, 2.5 g KH_2_PO_4_, 1.0 g NH_4_Cl, 0.1 g MgSO_4_·7H_2_O, 10 μL CaCl_2_ solution (745 g/L), 10 μL FeSO_4_ solution (265 g/L), and then add 1 mL Bauchop &Elsden solution (32). Bauchop &Elsden solution (1L):10.75 g MgO, 2.0 g CaCO_3_, 4.5 g FeSO_4_·7H_2_O, 1.44 g ZnSO_4_·7H_2_O, 1.12 g MnSO_4_·4H_2_O, 51.3 mL of saturated HCl solution. Minimal medium-citrate (MMC) was used for the KT2440[pPahpc] test by adding 20 mM (final concentration) of potassium citrate tribasic monohydrate to the minimal medium. Sodium chloride solution (0.85% [wt/vol]) was used for cell preservation. MgSO_4_·7H_2_O, FeSO_4_ were obtained from Aladdin (Shanghai, China), and the others were obtained from Sinopharm (Shanghai, China). All stock solutions of chemical reagents were filter-sterilized with 0.22 μm syringe filters (Millipore, USA).

### KT2440[pPahpc] responses to H_2_O_2_

The *P. putida* KT2440[pPahpc] strain was generously provided by Shanghai Jiao Tong University (31). Cultivation of KT2440[pPahpc] was performed in LB medium supplemented with tetracycline hydrochloride (50 μg/mL final concentration) under shaking (150 rpm) at 30°C for 16 h. Then, the KT2440[pPahpc] cells were harvested by centrifugation at 3,000 rpm for 10 min at 4°C. The cell pellets were washed three times with deionized water and finally suspended in half volume of 0.85% NaCl solution to produce the biosensor stock solution.

A 4 mM inducer stock solution was formulated by diluting 30% H_2_O_2_ in deionized water. The inducer stock solution was diluted to 0, 2.5, 5, 10, 25, 50, 100, and 500 μM with deionized water. A volume of 100 μL of inducer was added to the 96-well microplates, followed by 20 μL of biosensor stock solution and 80 μL of LB or MMC in a black, clear-bottom 96-well microplate (ThermoFisher, USA). All inducer concentrations were tested in triplicate, with deionized water serving as a negative control in place of the inducer. The 96-well microplate was then placed in a Tecan Spark microplate reader platform (Tecan Austria GmbH, Switzerland) and was incubated at 30°C with detection of luminescence and OD600 at 600 nm. Readings were taken every 5 min for 120 min, with 5 s of linear shaking prior to each reading to improve cell suspension.

ADPWH_salA containing the report gene *luxCDABE* (33) and the *P. putida* KT2440 wild-type strain, serving as the host for P*ahpc,* were incubated in LB at 30°Covernight. Next, 100 μL of bacterial culture and 100 μL of H_2_O_2_ solution were added to the 96-well plates to detect OD600 and luminescence.

### GluBio, UABio, and CholBio Assay Kit responses to glucose, uric acid, and cholesterol

The effectsof oxidase amount on the performance of the KT2440[pPahpc] were assessed using 0.2, 1,4,and 10 U of GOD, 0.1, 0.2,0.5, and 1 U of uricase, and COD. We prepared saturated aqueous uric acid solutions and quantified their concentrations using a UA quantification kit (BC160, Solarbio, China) following the manufacturer’s protocol (Fig. S4). A 1 M glucose stock solution and 1.3 mM cholesterol stock solution were simultaneously prepared for experimental use. The glucose solution stock was then diluted to final concentrations of 0, 5, 10, 20, 40,50, 100, and 200 μM. Saturated uric acid solution was diluted to final concentrations of 0,1.25,2.5,5,10,20,50, and 125 μM. The cholesterol stock solution was diluted to final concentrations of 0,1.25,2.5,5,10,20, and 100 μM. The biosensor working solution is prepared by mixing the biosensor stock solution with MMC medium in a ratio of 1:3. For the GluBio/CholBio assays, 80 μL of biosensor working solution was transferred to each well of 96-well plates, followed by adding 20 μL of glucose/cholesterol and 100 μL of GOD (0.2,1,4,and 10 U) or COD (0.1,0.2,0.5,and 1 U). For UABio Assay Kit, 80 μL of biosensor working solution was dispensed into 96-well microplates, followed by sequential addition of 100 μL uric acid standards and 20 μL uricase (0.1,0.2,0.5,and 1 U). Signal acquisition was carried out on a Tecan Spark microplate reader under the same operational settings previously optimized for KT2440[pPahpc] H_2_O_2_ detection assays.

### Real sample detection with GluBio and UABio Assay Kit

Fasting urine samples were collected from healthy volunteers (*n*=2; male and female), from whom informed consent was obtained. As endogenous H_2_O_2_ is naturally present in fresh human urine (34), two experimental groups were established. We measured glucose-derived H_2_O_2_ by comparing it to the baseline H_2_O_2_ level in 100-fold diluted urine. For the baseline, we combined 80 μL of biosensor working solution with 20 μL of 100-fold diluted urine and 100 μL of sterile deionized water in a microplate. In parallel, we mixed 80 μL of biosensor working solution with 20 μL of 100-fold diluted urine and 100 μL of GOD (1 U/reaction). Luminescence signals were recorded from both for quantification. We measured uric acid-derived H_2_O_2_ by first establishing a baseline with a mixture containing 80 μL of biosensor working solution, 100 μL of 250-fold diluted urine, and 20 μL of sterilized deionized water. For the quantification reaction, we then combined 80 μL of the biosensor working solution with 100 μL of 250-fold diluted urine and 20 μL of uricase (0.2 U/reaction). The signal from this reaction corresponds to the sum of endogenous and uricase-generated H_2_O_2_.

Urine specimens (*n*=17) were collected from the Department of Endocrinology and Metabolism of the Fourth Affiliated Hospital of Soochow University, comprising 13 samples from diabetes mellitus inpatients (ID 1–13) and 4 non-diabetic controls (ID 14–17). The clinical samples used in this study were collected as part of a larger research project funded by the National Natural Science Foundation ofChina (no.82270755), which was officially launched in 2022. Our current study, which focuses on H_2_O_2_ biosensors for the detection of multiple analytes, constitutes a sub-project within this approved framework, and all sample collection and handling procedures strictly adhered to the protocols outlined in the original 2022 ethics approval. Glucose and uric acid levels were quantified using our GluBio and UABio Assay Kits following the established protocols. For method validation, parallel measurements were conducted with commercial kits (glucose: Solarbio BC8322; uric acid: Solarbio BC1365) according to the manufacturer’s specifications, enabling direct comparison of biosensor performance against standardized assays.

## RESULTS

### Detection of multiple small molecules using an H_2_O_2_ whole-cell biosensor

*P. putida* KT2440[pPahpc] has been developed previously to express *lux*CDABE in response to H_2_O_2_ (31). First, we evaluated the concentration-dependent response range and limit of detection (LoD) of the KT2440[pPahpc] biosensor to H_2_O_2_. The KT2440[pPahpc] biosensor demonstrated a linear response for H_2_O_2_ detection within the range of 5–500 μM (Fig. S1A through C) when cultured in LB medium, achieving anLoD of 10 μM under standardized assay conditions. The salicylate biosensor ADPWH_salA, which harbors the identical reporter gene *lux*CDABE (33) as the H_2_O_2_ biosensor, was employed as a control to determine whether the reporter gene itself could be induced by H_2_O_2_. The results indicate that ADPWH_salA did not respond to H_2_O_2_ treatment, suggesting that H_2_O_2_ does not induce leaky expression of *lux*CDABE (Fig. S1D). Moreover, we tested the response of the wild-type *P. putida* strain to hydrogen peroxide and found that it likewise did not induce luminescence (Fig. S1D), indicating high specificity of *P. putida* KT2440[pPahpc] response to H_2_O_2_.

Subsequently, three enzymatic coupling systems were developed by combining KT2440[pPahpc] with specific oxidases: (i) GOD for glucose assay (GluBio), (ii) uricase for uric acid assay (UABio), and (iii) COD for cholesterol assay (CholBio) (Fig. 1 andTable 1). Initial characterization of KT2440[pPahpc] biosensors cultured in LB medium revealed elevated baseline luminescence across two assay platforms (GluBio, UABio; Fig. S2). To overcome the signal-to-noise limitations, we employed a carbon source optimization strategy by shifting to minimal media supplemented with systematically varied concentrations (10, 20, and 40 mM) of glycerol, citrate, succinate, and arabinose. After monitoring the kinetics for growth over 24 h, biosensors cultivated in minimal medium enriched with 20 mM citrate exhibited dual benefits: enhanced growth and minimized bioluminescence background (Fig. S3). Consequently, this optimized citrate-based minimal medium (termed MMC) was established as the standardized culture condition for all subsequent biosensing applications.

**TABLE 1:** Strains and assay kits for glucose, uric acid, and cholesterol in this study

Bacterial strain or kit name	Description	Source
Strains		
KT2440[p*Pahpc*]	*P. putida* KT2440 containing the *ahpc*promoter, which can sense H_2_O_2_	31
ADPWH_salA	*Acinetobacter baylyi* ADP1 with an integrated*salA* promoter-*lux*CDABE construct	33
*P. putida* KT2440	*P. putida* KT2440 wild-type strain	
Kits		
GluBio Assay Kit	KT2440[p*Pahpc*] biosensor coupled with glucose oxidase, which can sense glucose	This study
UABio Assay Kit	KT2440[p*Pahpc*] biosensor coupled with uricase, which can sense uric acid	This study
CholBio Assay Kit	KT2440[p*Pahpc*] biosensor coupled with cholesterol oxidase, which can sense cholesterol	This study

**Fig 2:**
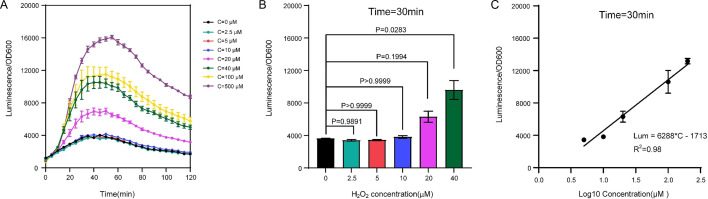
H_2_O_2_ detection by KT2440[p*Pahpc*]. (**A**)Kinetic response of KT2440[p*Pahpc*] to different concentrations of H_2_O_2_ induction(0–500 μM). (**B**)Luminescence/OD600 after 30 min exposure to H_2_O_2_(0–40 μM). Statistically significant differences were determined by the Kruskal–Wallis test (*P* < 0.05). (**C**)Luminescence/OD600 response of KT2440[p*Pahpc*] as a function of log_10_ (concentration) after 30 min induction. Luminescence signals (mean ± S.E.M., *n* = 9) were recorded at 5min intervals over a 2h period. The solid line shows the linear regression fit.

The KT2440[pPahpc] biosensor exhibited a linear H_2_O_2_ detection range of 20–500 μM in MMC (Fig. 2A). Upon induction with 40 μM H_2_O_2_, the luminescence per OD600 (Lum/OD) reached 9,600 ± 1,300, marking a 2.7-fold increase compared to baseline levels (3,636 ± 29 Lum/OD; *P*<0.05). A linear relationship was established between the logarithmic H_2_O_2_ concentration and luminescence per OD unit after 30min induction, which can be mathematically expressed as Lum/OD = 6,288 × *C*(μM) − 1,713 (*R*² = 0.98), where Lum/OD is luminescence/OD600, and *C* represents the logarithmic H_2_O_2_ concentration in μM (mean ± SD). This linear response range empowered KT2440[pPahpc] to deliver semi-quantitative measurements of H_2_O_2_ levels, serving as a robust foundation for dependable downstream detection in GluBio, UABio, and CholBio assays.

### Optimization of the GluBio, UABio, and CholBio Assay Kits for detecting glucose, uric acid, and cholesterol

To determine the optimal enzyme concentrations for the GluBio, UABio, and CholBio assays, dose-response experiments were conducted with GOD (0.2, 1, 4, and 10 U), uricase (0.1, 0.2, 0.5, and 1 U), and COD (0.1, 0.2, 0.5, and 1 U), which were coupled with KT2440[pPahpc] for verification of concentrations of glucose, uric acid, and cholesterol dynamics respectively.

**Fig 3:**
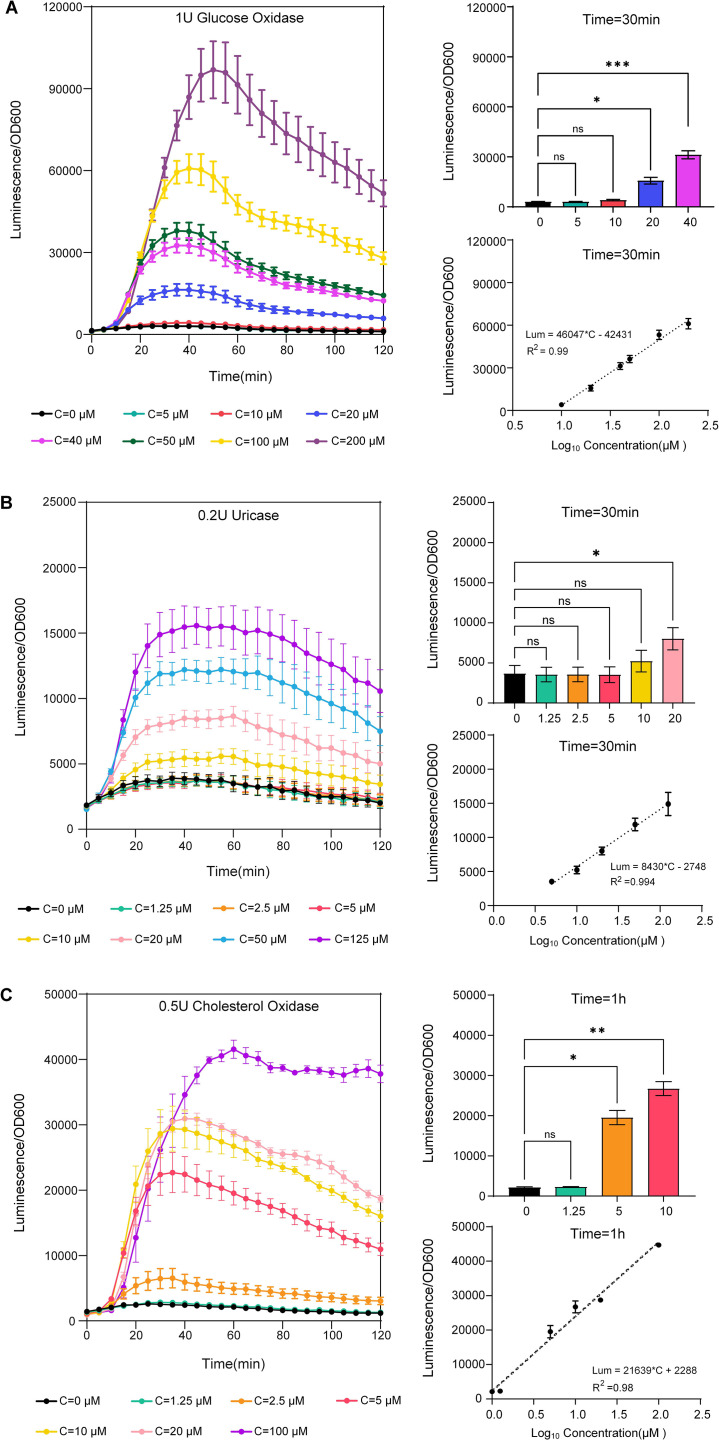
Results of GluBio, UABio, and CholBio assays. (**A–C**)Lum/OD vs log_10_ (analyte concentration) for glucose (1–200 μM), uric acid (0–125 μM), and cholesterol (0–100 μM). Enzymes (glucose oxidase: 1 U, uricase: 0.2 U, cholesterol oxidase: 0.5 U) were added at *t*=0. The *P* values determined by one-way ANOVA followed by Kruskal-Wallis test for statistical comparison of luminescence across concentrations for glucose(**A**), uric acid(**B**), and cholesterol (**C**)were *P<0.05, **P <0.01, and ***P <0.001. All the data were shown as mean ± S.E.M. (*n*=9). Luminescence was captured every 5 min for 2 h.

For glucose detection using the GluBio assay, a linear correlation was observed between the Luminescence/OD600 and the logarithmic concentration of glucose over the range of 0–200 μM when supplemented with 1 U of GOD (*R*^2^ = 0.99; Fig. S5A). The regression equation was Lum/OD=46,047 × *C* (μM) −42,431 (Lum/OD is the luminescence/OD600, and *C* is the logarithmic glucose concentration), showing that GluBio Assay Kit could be used for quantification of the glucose concentration. The LoD of the GluBio Assay Kit for glucose was 20 μM Fig. 3A).

For uric acid detection using the UABio assay, a linear fit was observed between the Lum/OD and the logarithmic concentration of uric acid in the range of 0–125 μM when supplemented with 0.2 U (*R*^2^ = 0.99) (Fig. S5B). The regression equation was Lum/OD= 8,430 × *C*(μM)−2,748 (Lum/OD is the luminescence/OD600, and *C* is the logarithmic uric acid concentration) (Fig. 3B). The LoD of the UABio Assay Kit for uric acid was 10 μM (Fig. 3B).

For cholesterol detection using the CholBio assay, the biosensor exhibited a linear response within 15 min across the concentration of 2.5–100 μM (Fig. 3C) when supplemented with 0.5 U (Fig. S5C). The regression equation was Lum/OD=21,639 × *C*(μM) +2,288 (*R*^2^ = 0.98), where Lum/OD is the luminescence/OD600, *C* is the logarithmic cholesterol concentration (Fig. 3C). The LoD of the CholBio Assay Kit for cholesterol was 5 μM.

### Glucose, uric acid, and cholesterol quantification with alternative H_2_O_2_ detection approaches

To verify the kinetics of target substrate oxidation to H_2_O_2_, a standardized fluorescent-based H_2_O_2_ assay (MAK165-1KT, Sigma-Aldrich) was performed with each of the three enzymes individually. Dose-response analyses revealed a distinct linear relationship between the fluorescence intensity and the substrate concentrations, with detection ranges of 0–2.5 μM for glucose (*R*²=0.994), 0–25 μM for uric acid (*R*²=0.972), and 0–5 μM for cholesterol (*R*²=0.986) (Fig. S6A through C). These results confirmed the feasibility of determining the concentrations of glucose, uric acid, and cholesterol by measuring H_2_O_2_ levels using the biosensor.

### Semi-quantification of glucose and uric acid in urine with the GluBio and UABio Assay Kits

**Fig 4:**
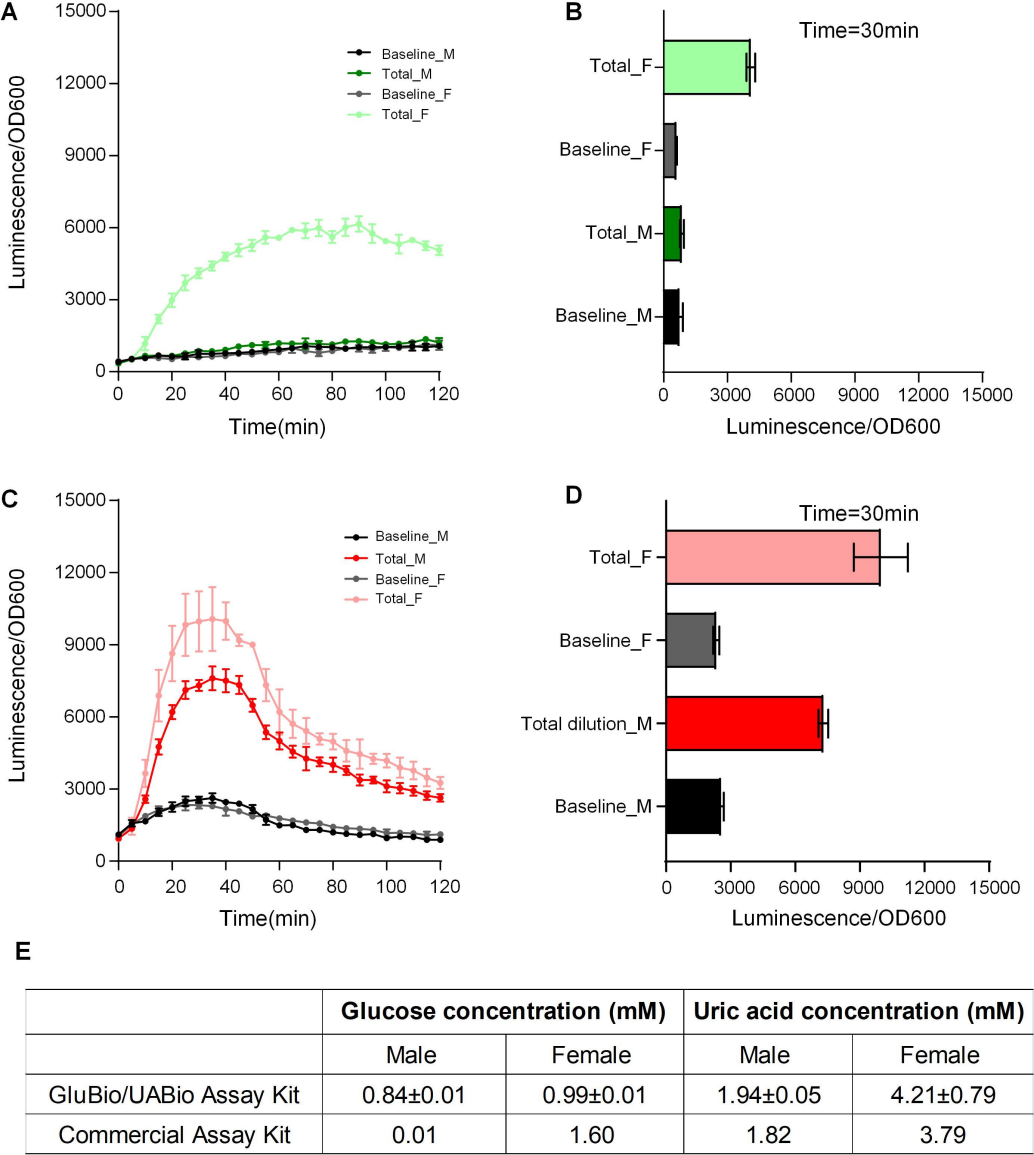
Glucose and uric acid detection in urine using GluBio and UABio assays. (**A**)Kinetics of glucose detection with GluBio Assay Kit in urine.Glucose oxidase (1 U) was added into the reaction at time zero. (**B**)The Lum/OD reads for glucose detection in urine after 30 min. (**C**)Kinetics of uric acid detection with UABio Assay Kit in urine. Uricase (0.2 U) was added into the reaction at time zero. (**D**)The Lum/OD reads for uric acid detection in urine after 30 min. (**E**)Calibrated concentrations of glucose and uric acid in urine. Total_F/M: Lum/OD of female/male urine with the corresponding oxidases added. Baseline_F/M: Lum/OD of female/male urines without related oxidases added.

To assess the clinical feasibility, the GluBio assay was validated using human urine specimens (*n*=2 volunteers). Kinetic luminescence analysis (Fig. 4A and B) yielded mean glucose concentrations of 0.84 and 0.99 mM in 30min induced samples, calculated via the pre-established calibration curve (Lum/OD=46,047 × *C*(μM)−42,431, *R*²=0.99) (Fig. 3A). Both values fell within the normal reference interval (Fig. 4E). For uric acid detection with the UABio assay, the kinetic curves for male and female urine samples were similar (Fig. 4C). After 30 min, the total value of luminescence induced by female urine was higher than that of male urine (Fig. 4D). Using the formula Lum/OD=8,430 × *C*(μM)−2,748(Fig. 3B), the calculated uric acid concentration in male urine and female urine was approximately 1.94 and 4.21 mM, respectively (Fig. 4E).

Validation using a commercial glucose/uric acid assay kit yielded concentrations of 0.01 mM (male) and 1.6 mM (female) for urinary glucose, and 1.82 mM (male)and 3.79 mM (female) for uric acid (Fig. 4E). These results demonstrated strong concordance with measurements obtained via the biosensor-based assays, confirming the GluBio/UABio kits’ capacity for clinical-grade biomarker quantification in human urine specimens.

### Pre-clinical trial using GluBio and UABio assays

Based on the preliminary detection results, we further assessed the clinical utility of the GluBio and UABio Assay Kits by analyzing glucose and uric acid concentrations from 17 clinical urine samples collected from The Fourth Affiliated Hospital of SooChow University (Suzhou, China). It is worth noting that all the samples were collected from hospitalized patients. Samples 1–13 were from diabetic patients, while samples 14–17 were from non-diabetic patients. Diabetic patients corresponding to samples 1–13 were receiving treatment, including medications to lower blood glucose levels during their hospitalization, which may have affected urinary glucose concentrations.

**Fig 5:**
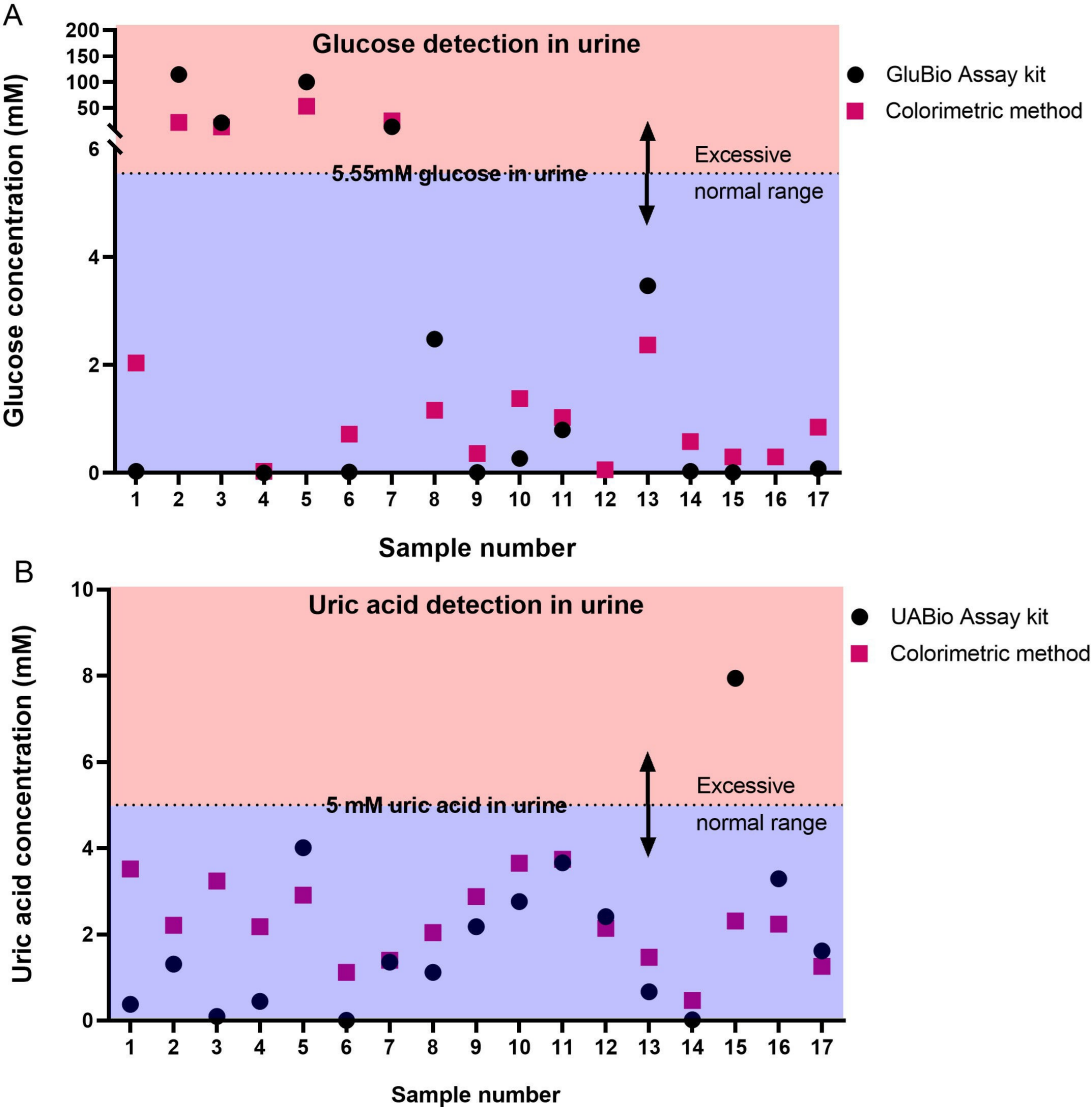
Clinical validation of GluBio and UABio assays in urine samples.(**A**)The results of the GluBio Assay Kit and the colorimetric method for detecting glucose concentration in urine showed 100% concordance (17/17 samples). Dashed line: normal urinary glucose threshold (5.55 mM). Samples 2, 3, 5, and 7 exceeded this value, indicating potential diabetes risks.(**B**)The UABio assay and conventional luminescence assay achieved 94% concordance (16/17 samples). Dashed line: normal urinary uric acid threshold (5 mM).

For glucose quantification in urine samples from diabetic patients, based on the Fulin-Wu chemical colorimetric method (commercial kit), reported concentrations of 22.42 mM (patient no.2), 53.41 mM (patient no.5), and 25.29 mM (patient no.7). Given that the GluBio kit’s linear detection range is 10–200μM and the normal urine glucose levels are typically below 5.55 mM (35), these samples were diluted 250-fold to align with the assay’s measurable range. The GluBio kit successfully confirmed that the glucose concentrations in these patients’ urine samples exceeded 20 mM (Fig. 5A). In addition, the glucose concentrations in urine samples of no. 4, no. 6, no. 9, no. 11, and no. 12 were measured to be below 2 mM using the commercial kit, which were consistent with the results from the GluBio kit (Fig. 5A). For non-diabetic patients, both the commercial kit and the GluBio kit were consistent, reporting the urinary glucose concentrations below 2 mM. The detailed information is shown in Table S2.

For the detection of uric acid concentration in urine samples, we also compared the commercial kit with the UA kit. The UA kit has a linear detection range of 5–125 µM, and considering that urinary uric acid concentrations are typically below 5 mM (36), we diluted all urine samples 250-fold for the tests. The results obtained from the commercial kit and our UABio kit demonstrated consistency for samples no. 1–14 and 16–17, with urinary uric acid concentrations remaining within the normal range (<5mM) in both analyses(Fig. 5B). The sole exception was sample no. 15, which showed divergent results between the two methods. Our kit flagged its uric acid concentrations as abnormal, whereas the chemical colorimetric assay reported values within the normal range (Fig. 5B). This discrepancy could be attributed to urine-borne interferents in this patient’s sample, which may have compromised biosensor specificity or signal stability.

## DISCUSSION

Traditional whole-cell biosensors are typically engineered for single-substrate detection to maintain analytical specificity (37, 38). Consequently, conventional approaches necessitate the labor-intensive development of distinct biosensors for different target substances—a process that proves both time-consuming and cost-consuming for practical applications (39). To address this limitation, we present an innovative multiplex detection system utilizing a H_2_O_2_-responsive whole-cell biosensor capable of sequentially analyzing glucose, uric acid, and cholesterol. This methodological advancement exploits H_2_O_2_ as a universal signaling molecule generated through enzymatic oxidation of specific substrates. By employing a single biosensor platform coupling with multiple enzyme systems to detect different analytes, the time and cost associated with sensor design and validation can be significantly reduced. Notably, this approach enables convenient and rapid biosensing even for chemical substances lacking precise regulatory systems. Consequently, this method is broadly applicable across various fields, including food safety, environmental monitoring, and clinical diagnostics.

Urine, widely recognized for its non-invasive and accessible collection (40), serves as a prime diagnostic fluid enriched with disease-relevant metabolites such as glucose and uric acid—key biomarkers in clinical diagnostics. In this study, we assessed the GluBio and UABio Assay Kits for quantifying these urinary biomarkers. Notably, cholesterol analysis was excluded from urine testing due to its physiological absence or crystallization in urine under normal conditions (41). Due to the inherent complexity of whole blood, which contains numerous cellular components, direct detection using biosensors remains challenging. Consequently, we did not test our biosensor with whole-blood samples in this study. For future work, we will focus on developing simple pretreatment methods to prepare blood samples for biosensing, aiming to achieve broader applicability across various sample types.

The platform exhibited clinically meaningful sensitivity, with LoD of <20µM for glucose, <10µM for uric acid, and <5µM for cholesterol—all surpassing pathological thresholds in human urine. Prior studies reported physiological baselines for healthy individuals as follows: blood glucose < 6.9mM (42), urine glucose <5.55mM (35), blood/urine uric acid <0.453/5.0 mM (36), and blood cholesterol < 5.2mM (43) (Table S1). Our developed biosensing assays achieved clinically significant sensitivity, with LoD 200–1,000-fold below these baselines across all targets. This ultrasensitive detection empowers precise monitoring of disease biomarkers, including glucose (diabetes biomarker) and uric acid (gout indicator), while maintaining robust linear correlations (*R*² = 0.98–1.00) across calibration ranges. Additionally, the response time of less than 30 min enables point-of-care testing in both home and clinical settings. The urine samples we collected from the hospital were obtained from hospitalized patients undergoing treatment. Due to the extensive use of multiple medications, the composition of these urine samples is notably more complex. During collection, we observed that some urine samples exhibited chylous turbidity; however, testing with our developed kit demonstrated that accurate detection was still achievable, further confirming the robustness of our assay. In real urine samples, due to the complexity of urine specimens, results from GluBio and UABio assays are more suited to qualitative assessment in home settings, such as determining whether glucose and uric acid levels fall within normal ranges. Moreover, in the future, simple urine pretreatment reagents or devices could be developed to minimize interferents in samples, thereby enhancing detection accuracy.

The use of the H_2_O_2_-based biosensor for detecting three diseases in this study represents a proof-of-concept, showcasing broader applicability in diverse fields such as medical diagnostics, food safety, and environmental monitoring, avoiding the time and cost of developing multiple sensors. Emerging evidence highlights the prospective integration of this biosensing technology with smart sanitation systems, enabling real-time, multi-parameter health monitoring via routine excretion. This transformative approach enables continuous collection of longitudinal physiological data, which can be analyzed by AI-driven decision support systems to deliver personalized interventions—including tailored nutrition, optimized pharmacotherapy, and proactive health strategies.

## SUPPLEMENTAL MATERIAL

Supplemental material aem.00338-26-s0001.docx Fig. S1 to S6; Tables S1 and S2.

ASM does not own the copyrights to Supplemental Material that may be linked to, or accessed through, an article. The authors have granted ASM a non-exclusive, world-wide license to publish the Supplemental Material files. Please contact the corresponding author directly for reuse.
