# Supplementary material for: Integration of substrate-specific enzymes and a peroxide biosensor for detection of glucose, uric acid, and cholesterol
Source: Appl Environ Microbiol. 2026 May 18;92(6):e00338-26. doi: 10.1128/aem.00338-26 (PMC13274428; doi:10.1128/aem.00338-26)
Supplement: Supplemental material — Fig. S1 to S6; Tables S1 and S2. [file aem.00338-26-s0001.docx]

**The integration of substrate-specific enzymes and a peroxide biosensor to the detection of glucose, uric acid and cholesterol**

**Detection of glucose, uric acid and cholesterol with fluorometric assay kit**

We used a fluorimetric hydrogen peroxide assay kit (MAK165,Sigma, USA) to quantify H_2_O_2_ production from glucose, uric acid and cholesterol catalyzed by specific oxidases. For glucose detection, H_2_O_2_ standards (20 mM stock) were diluted to final concentrations of 0.01,0.03,1 and 10 μM, while glucose solutions (1M stock) were diluted to 0.25, 0.5, 1,2.5, 5,10 μM. A master mix was prepared by combining 50 μL of red peroxidase substrate, 20 units/ml of peroxidase stock, and 4.75 mL of assay buffer for a 96-well plate. Finally, 50 μL of the master mix and 50 μL of H_2_O_2_ were added to each well as the standard group, and 50 μL master mix, 10 μL glucose and 40 μL GOD (1U) were added to each well as the sample group. After 15 minutes of room temperature incubation, fluorescence was measured at lex=540/lem=590 using a Tecan spark plate reader.

For uric acid detection, the 20 mM H_2_O_2_ standard was diluted to final concentrations of 2.5,5,10,20,40 and 50 μM and uric acid solutions were diluted to 1.25,2.5,5,10,20 and 25 μM. The formula of the master mix and standard group was the same as described above. Fifty microlitres of the master mix, 10 μL of uric acid and 40 μL of uricase (0.2U) were added into each of the wells as the sample group. For cholesterol detection, the 20 mM H_2_O_2_ standard was diluted to 1.25,2.5,5 and 20 μM. The formula of the master mix and standard group was the same as above. Samples wells were prepared with 50 μL master mix, 10 μL cholesterol and 40 μL COD (0.5U). Fluorescence was measured as described above.


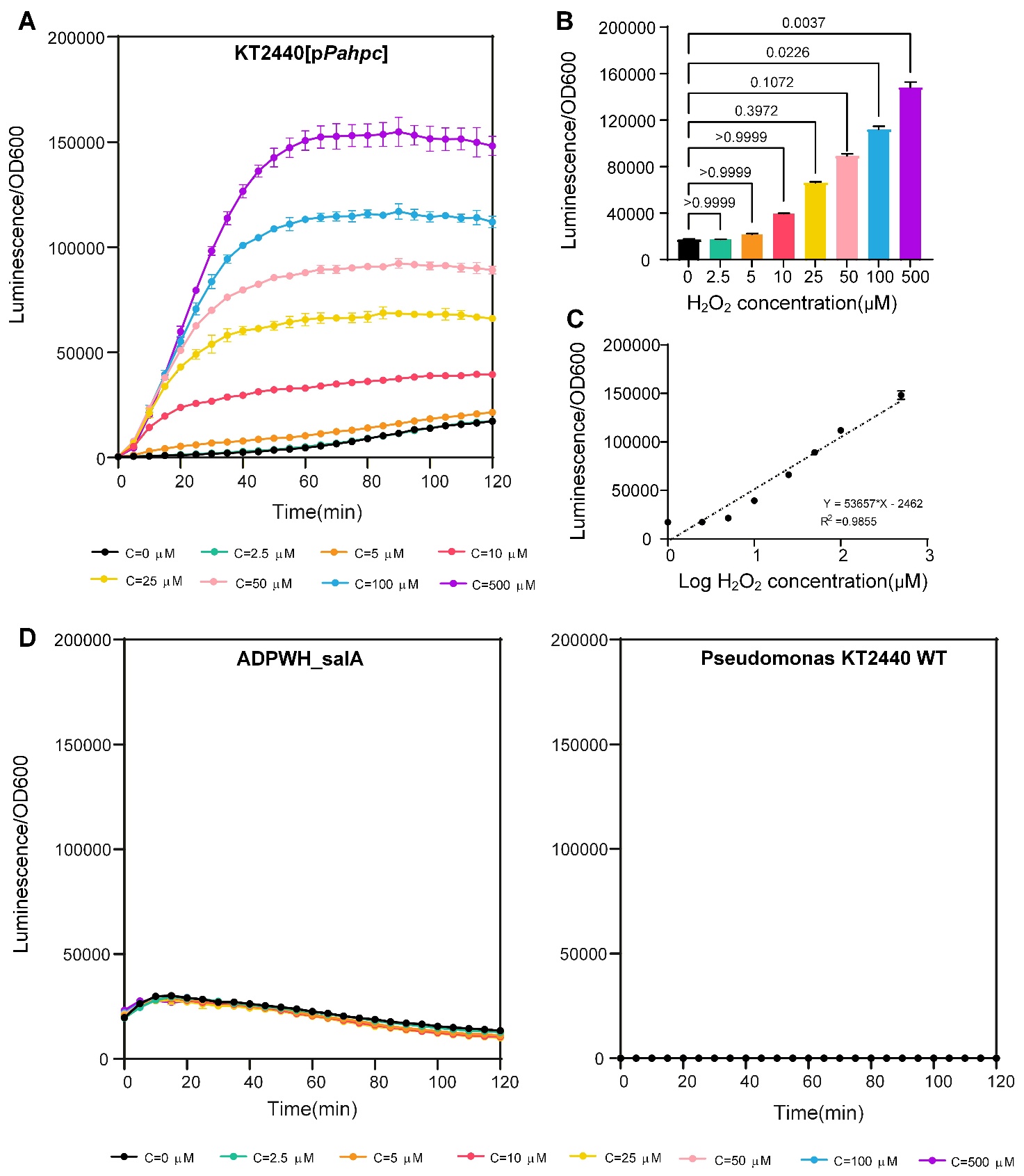


**Fig.S1.** Response of KT2440[p*Pahpc*] for H_2_O_2_ induction. (A)Dose-response kinetics of KT2440[p*Pahpc*] in LB with H_2_O_2_ induction gradients (0-500 μM). (B) Lum/OD at 30 min exposure to H_2_O_2_ (0-10 μM), with significant differences (*P< 0.05, Kruskal-Wallis test). (C) Linear correlation between Lum/OD and logarithmic H_2_O_2_ concentration after 30 min induction. Kinetic profiles were captured every 5 min over 2 hours. (D) ADPWH_salA and *P.putida* KT2440 wide type response for H_2_O_2_, results are representative of biosensor control for H_2_O_2_ induction. The data in A, **B**, **C**, **D** are shown as Mean$\pm$S.D. (n=3).


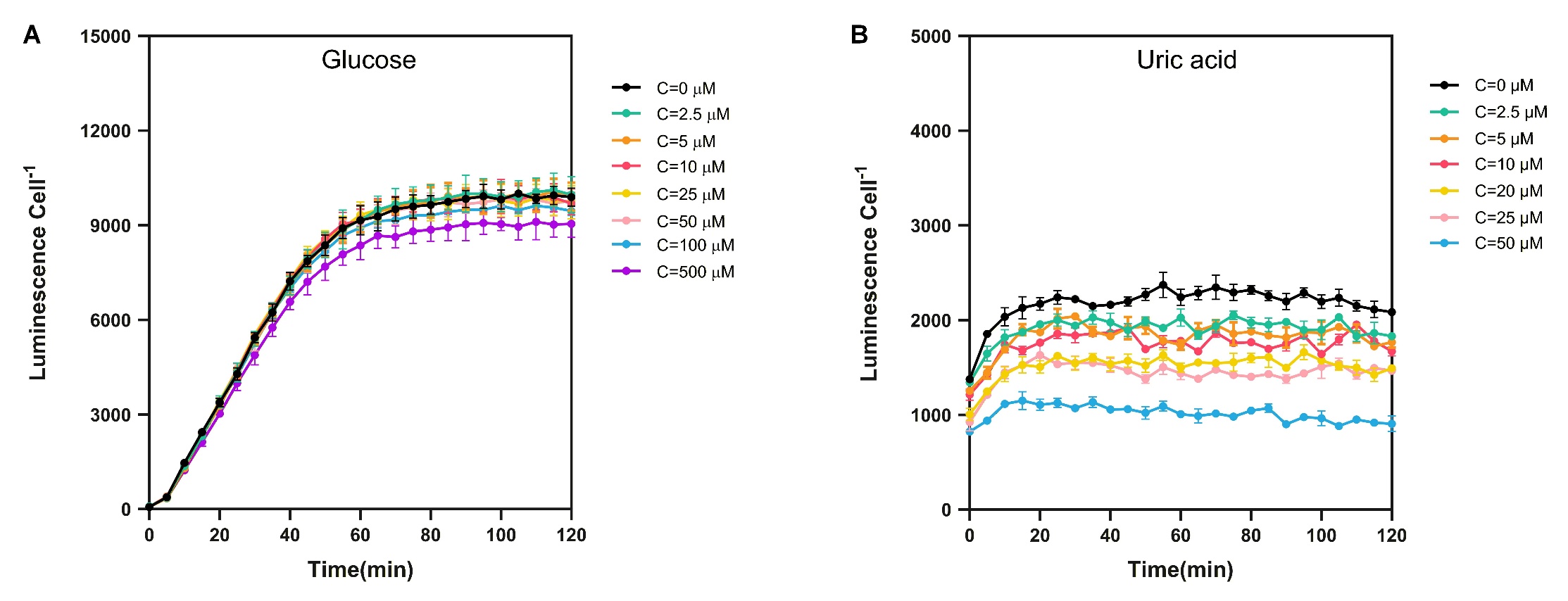


**Fig.S2.** Luminescence Cell^-1^ response curves of the GluBio Assay Kit for glucose (A) and UABio Assay Kit for uric acid (B) detection in LB medium as the nutritional matrix respectively. No significant luminescence response was observed over glucose concentrations of 0–500 μM (A) or uric acid concentrations of 0–50 μM (B).


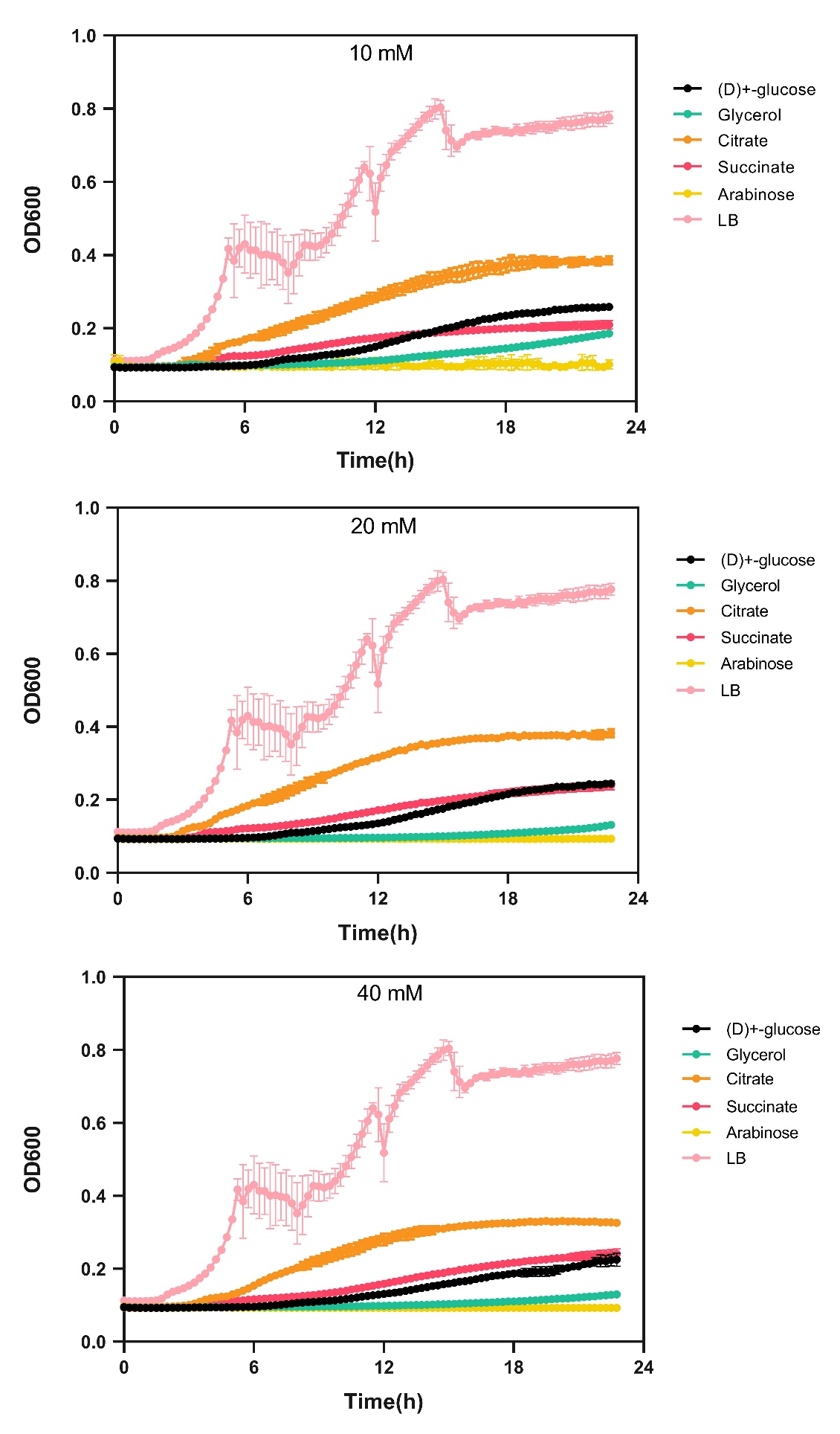


**Fig.S3.** Selection of carbon source for KT2440[p*Pahpc*]. Minimal medium was supplemented with 10 mM, 20 mM and 40 mM of (D)+-glucose, glycerol, citrate, succinate and arabinose individually. The absorbance of 600 nm (OD600) was obtained every 15 min for 24 hours.

**Fig.S4.** The concentration of saturated uric acid solution was determined using the uric acid detection kit (BC1360). Absorbance at 505 nm was measured, with △Absorbance calculated as A505 (substrate) - A505(Blank).


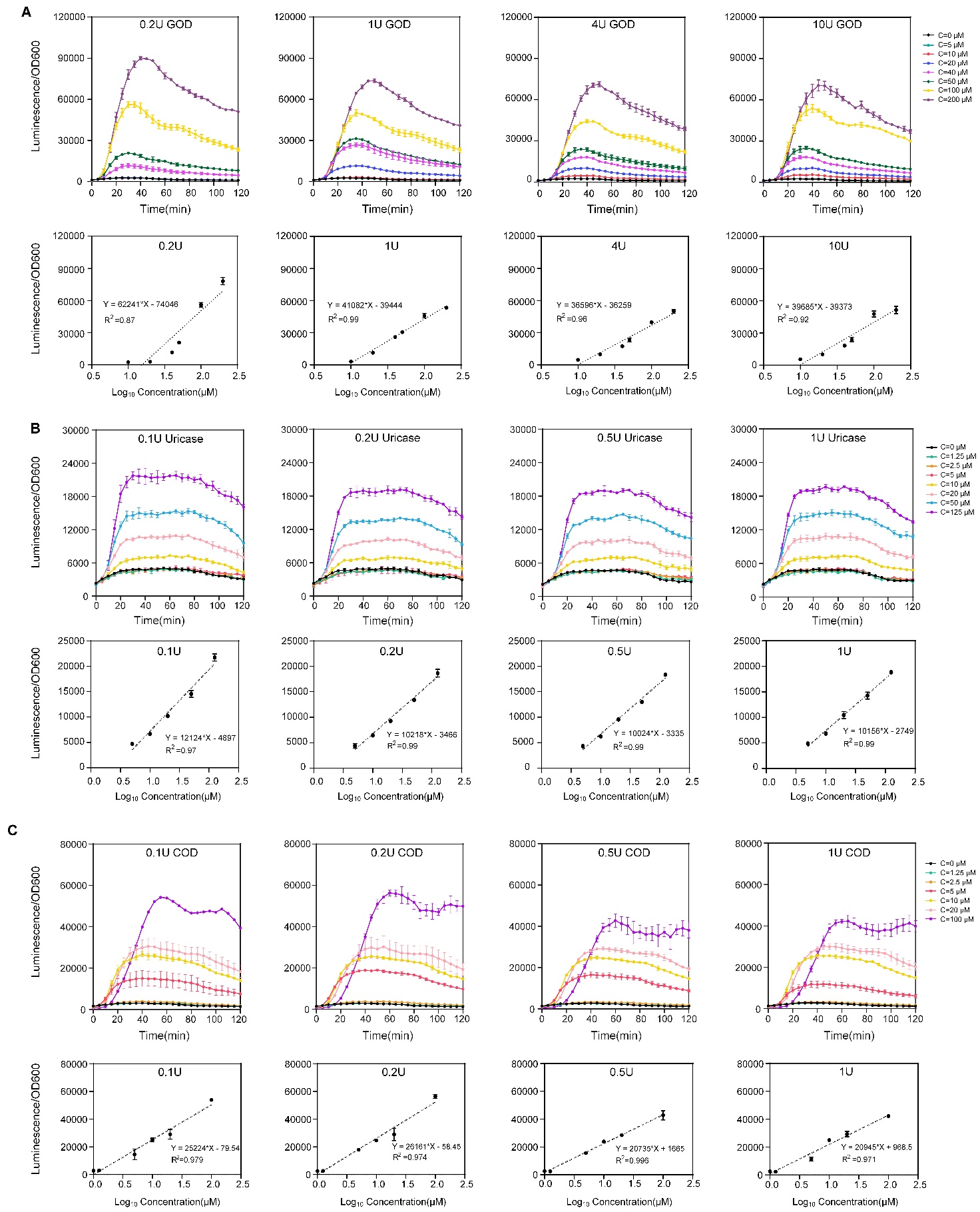


**Fig.S5.** Effect of oxidase amount on KT2440[p*Pahpc*] for detecting glucose(A), uric acid(B) and cholesterol(C). (A) Four glucose oxidase doses (0.2U,1U, 4U and 10U) were tested. A linear correlation between Lum/OD and logarithmic glucose concentration was established after 30 min induction. (B) Four doses of uricase (0.1U,0.2U, 0.5U and 1U) were tested. A linear correlation between Lum/OD and logarithmic uric acid concentration was established after 30 min induction. (C) Four doses of cholesterol oxidase (0.1U,0.2U and 0.5U and 1U) were tested. A linear correlation between Lum/OD and logarithmic cholesterol concentration was established after 1 h induction. Luminescence was monitored every 5 min over 2 hours. Data are presented as Mean ± S.D. (n=3).


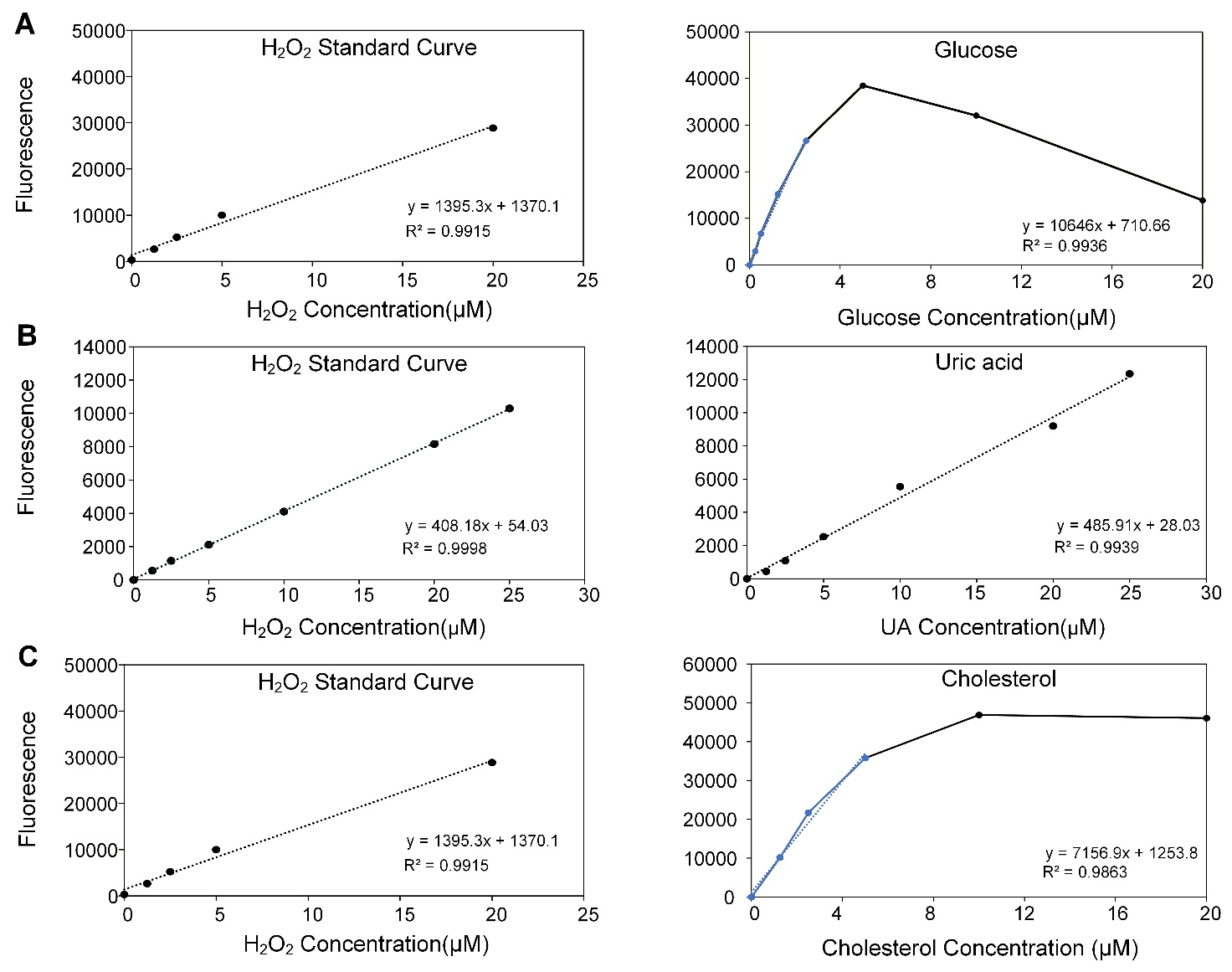


**Fig.S6.** Standard curve for H_2_O_2_ detection using fluorometric hydrogen peroxide assay kit. **(**A) Fluorescence as a function of the H_2_O_2_ concentration, and the linear response at different concentration of H_2_O_2_. The standard sample gradient was 0,1.25,2.5,5,20 μM. Fluorescence as a function of the glucose concentration, and the linear response at different concentration of glucose. The glucose gradient was 0,0.25,0.5,1.25,2.5,5, 10,20 μM. And 1U of oxidase was added into the reaction, the H_2_O_2_ and glucose were detected at the same time.(B) Fluorescence as a function of the H_2_O_2_ concentration, the gradient was 0,1.25,2.5,5,10,20,25 μM. Fluorescence as a function of the uric acid concentration, the gradient was 0,0.25,0.5,1,2.5,5,10 μM, and 0.2U of uricase was added into reaction, the H_2_O_2_ and uric acid were detected at the same time.(C) Fluorescence as a function of the H_2_O_2_ concentration, the gradient was 0,1.25,2.5,5,20 μM. Fluorescence as a function of the cholesterol concentration, the gradient was 0,1.25,2.5,5, 10,20 μM, and 0.5U of cholesterol oxidase was added into reaction, the H_2_O_2_ and cholesterol were detected at the same time. The fluorescence was obtained with lex = 540/lem = 590 nm. The data was shown as Mean±SD (n=3).

**Table.S1.** Concentrations of glucose, uric acid and cholesterol in physiological fluids of healthy human body and a volunteer in this study.

|  | Optimal range (mmol/L) | | Our study(mmol/L) |
| --- | --- | --- | --- |
| Physiological Fluid | In blood | In urine | In urine |
| Glucose | <6.9 | <5.55 | 0.92 |
| Uric acid | <0.45 | <5.0 | 3.07 |
| Cholesterol | <5.2 | \ |  |

**Table.S2.** Detection of glucose and uric acid in clinical urine samples using kits developed in this study.

| **Glucose detection in urine with GluBio Assay kit** | | | | | |
| --- | --- | --- | --- | --- | --- |
| **Sample number** | **Diabetes** | **Concentration(mM) based on GluBio Assay Kit** | | **Concentration（mM） based on** **Colorimetric method** | |
| 1 | Yes | ≤2 | | 2.04 | |
| 2 |  | ≥20 | | 22.42 | |
| 3 |  | ≥20 | | 13.57 | |
| 4 |  | ≤2 | | 0.03 | |
| 5 |  | ≥20 | | 53.41 | |
| 6 |  | ≤2 | | 0.72 | |
| 7 |  | ≥20 | | 25.29 | |
| 8 |  | 2.48 | | 1.16 | |
| 9 |  | ≤2 | | 0.36 | |
| 10 |  | ≤2 | | 1.38 | |
| 11 |  | ≤2 | | 1.03 | |
| 12 |  | ≤2 | | 0.06 | |
| 13 |  | 3.47 | | 2.37 | |
| 14 | No | ≤2 | | 0.58 | |
| 15 |  | ≤2 | | 0.30 | |
| 16 |  | ≤2 | | 0.30 | |
| 17 |  | ≤2 | | 0.85 | |
| **Uric acid detection in urine with UABio Assay Kit** | | | | | |
| **Sample number** | **Concentration(mM) based on UABio Assay Kit** | | **Concentration (mM) based on Colorimetric method** | |  |
| 1 | ≤1.25 | | 3.52 | |  |
| 2 | 1.31 | | 2.21 | |  |
| 3 | ≤1.25 | | 3.24 | |  |
| 4 | ≤1.25 | | 2.18 | |  |
| 5 | 4.01 | | 2.91 | |  |
| 6 | ≤1.25 | | 1.12 | |  |
| 7 | 1.36 | | 1.40 | |  |
| 8 | ≤1.25 | | 2.04 | |  |
| 9 | 2.18 | | 2.88 | |  |
| 10 | 2.76 | | 3.65 | |  |
| 11 | 3.66 | | 3.74 | |  |
| 12 | ≤1.25 | | 2.14 | |  |
| 13 | ≤1.25 | | 1.47 | |  |
| 14 | ≤1.25 | | 0.47 | |  |
| 15 | 7.94 | | 2.31 | |  |
| 16 | 3.29 | | 2.24 | |  |
| 17 | 1.62 | | 1.26 | |  |
